# Glocalization of bioethics

**DOI:** 10.1080/11287462.2022.2052603

**Published:** 2022-03-19

**Authors:** Himani Bhakuni

**Affiliations:** a University Medical Center Utrecht, Utrecht University, Utrecht, The Netherlands; b Department of Foundations and Methods of Law, Maastricht University, Maastricht, The Netherlands

**Keywords:** Glocalization, glocal bioethics, cultural difference, Lesbian rule, informed consent

## Abstract

There appears to be a conflict between global bioethical principles and the local understanding and application of these principles, but this conflict has misleadingly been characterized through the east–west dichotomy. This dichotomy portrays bioethical principles as western and as alien to non-western cultures. In this paper, I present reasons to reject the east–west dichotomy. Using the discussion around the principle of informed consent as an example, I propose that while bioethical values are common, bioethical governance must display a certain flexibility akin to Aristotle’s metaphor about the Lesbian rule. Such flexibility combined with a deeper understanding of the lived experiences of bioethical subjects might lead to the purging of tensions between global and local, giving us *Glocal Bioethics*.

## Introduction

The term “globalization” tends to be both ambiguous and value laden. It is a multidimensional concept that adapts to the blinders of the discipline it represents. Globalization has essentially been understood as either a homogenous or a heterogenous concept (Scholte, 2002). The homogenous version regards it as a process of integrating people around the world into a single world society (Pieterse, 1994), which has developed further into alternate ideas that either denote synchronization of consumption culture, cultural imperialism, Americanization, Westernization, or the “the power of transnational capitalism to distribute its cultural goods around the world” (Tomlinson, 1999). The heterogenous version focuses on locality or the place where people live in their daily lives and which is slow to adapt to the standardization and connectivity of the global (Tomlinson, 1999).

Glocalization is similar to the heterogenous reading of globalization. It is characterized in the interconnectedness of many local cultures; it “involve[s] the creation and the incorporation of locality, processes which themselves shape the compression of the world as a whole” (Robertson, 1995). It is widely accepted that glocalization has its origins in the Japanese idea of *dochakuka*, which was an agricultural principle of acclimating farming techniques to local conditions. The idea picked up academic usage in the 1990s with disciplines ranging from marketing to environmental studies developing techniques for cultural hybridization. The appropriation of “glocalization” is fluid, but the underlying idea of cultural hybridity and fusion is not entirely novel (Roudometof, 2015). Glocalization can be understood as an active process that does not unilaterally apply a single global culture around the world but that uncovers the ethos of different local contexts to find the global in it. It moves away from the imposing tendencies associated with globalization and makes way for a richer understanding of social processes.

In this paper, I advance a case for glocalization of bioethics by first giving an example of a much-debated principle from research ethics – informed consent – which often finds itself a part of the east–west dichotomy in bioethics. Through that example, I reject the east–west dichotomy which is represented through the “cultural-difference argument” and give reasons for why such a view of bioethical principles is misleading. I then explain the relevance of glocalization through Aristotle’s metaphor about the Lesbian rule and elucidate on what glocalization would look like in the bioethical arena. I also differentiate my notion of glocal bioethics with earlier proposals on what glocalization would mean for the field.

There is a widely held assumption that certain global bioethical challenges arise mainly due to the east–west cultural divide (Ekmekci & Arda, 2017; Raposo, 2019; Tan Kiak Min, 2017) instead of local socio-economic contexts. This assumption exemplifies a conflict between global and local – where global is widely understood to be western (or influenced by the west) and local is everything that is not. But proponents of the idea that global bioethics is an exercise in western moral/cultural imperialism (Chattopadhyay & De Vries, 2008 Garrafa & Lorenzo, 2008;) overlook the impact of non-western cultures on the west and on the interconnectedness of global and local, choosing instead to solely focus on the conflicts between global and local. The kind of glocalization that I propose in the latter half of this paper promises to remedy such conflicts, it attempts to give a solution to a problem – a problem that will be explained in detail through the principle of informed consent in the next section.

## Global/local and the east–west dichotomy

Several scholars have been sceptical of the idea that bioethics is truly global in the sense of employing universal principles. For example, informed consent is required to legitimize any intervention done on the human body and an intervention can only become legitimate when the essential requirements of informed consent are met. These essential requirements include voluntariness, adequate information disclosure, and capacity to consent (Faden & Beauchamp, 1986). Voluntariness is a highly debated concept in ethics and the idea that it requires autonomous decision-making has been central to many global/local debates that are characterized through the east–west dichotomy. Autonomous decision-making is contentious because many believe that autonomy, or the capacity of an individual to act freely, is a notion only applicable in the west and that such individualism is contrary to the family and community-oriented actions of people in the eastern nations. This gives rise to a long-running discussion in bioethics that suggests that principles are only local or regional expressions of a particular group of people and differ for different cultures, what Jing-Bao Nie calls the “cultural difference argument” (Nie, 2007). The argument rests on two assumptions. First, that a clear distinction can be drawn between the east and the west based on the values they prioritise. Second, that principles like informed consent that require autonomous decision-making exemplify western individualism and are inapplicable in the more communitarian and family-oriented east.

The cultural difference argument appeals to some scholars. For instance, when writing about informed consent as a principle, scholars like Schuman have argued that “[c]ultural differences between the First and Third Worlds further reveal that the informed consent standard is a uniquely Western concept, rather than a universal right appropriate for all societies” (Schuman, 2012). Others hold that “[t]he ethical principles of western countries require all adults to be the primary decision makers of their participation, which may not be applicable in [the] Indian system, which is culturally and socially different from the western world” (Nijhawan et al., 2013). The same attitude is held towards the principle of autonomy that is often used as the primary justification for informed consent. For instance, Chinese author Ruiping Fan holds the view that the western idea of autonomy, denoting individual independence, is incommensurable to the East Asian principle of autonomy, understood as family-oriented harmonious interdependence (Fan, 1997). Similar remarks have been made by Filipino authors, Alora and Lumitao, who state that “[w]estern ideals of individualism and self-reliance have little purchase in the Filipino culture” (Alora & Lumitao, 2001). In all these quotes the authors imply that principles of autonomy and informed consent might not be appropriate for some societies *because* they are western concepts and, as such, inapplicable in non-western societies.

One can see why the cultural difference argument could be appealing at first. The global or universal (or western) principle says that individuals should deploy autonomous decision-making while making decisions about their health, and a decision would only be autonomous if it is free from controlling influences (Nelson et al., 2011). But several cultural studies have shown that many people, particularly non-western societies, seem to defer to family or community while taking decisions that impact not only their health but also their lives in general (Coward & Sidhu, 2008; Letendre & Tham, 2011). It is understandable why some might reason that such fundamental differences in something as key to human life as decision-making has to do with not just cultural differences between the east and the west but also with fundamental differences in moral values. However, several objections can be raised against this view.

To elaborate on the first objection to the cultural difference argument let us consider Bernstein’s suggestion on incommensurability; he writes:

[W]e must always strive to avoid a false essentialism when we are trying to understand the traditions to which we belong or those alien traditions that are incommensurable with “our” traditions. For frequently discussions of East–West lapse into such a false essentialism where we are seduced into thinking there are essential determinate characteristics that distinguish the Western and Eastern “mind.” This false essentialism violently distorts the sheer complexity of overlapping traditions that cut across these artificial, simplistic global notions. (Bernstein, 2013)

Bernstein’s suggestion that those who write on the east–west divide lapse into wrong essentialism might have some weight. Wrong essentialism occurs when certain characteristics are attributed to everyone (or almost everyone) identified with a particular category, so generalizations like “Africans dance well”, “women are kind care givers”, or “Asians are community oriented” denote some degree of false essentialism. Norms, values, traditions have always found a way to communicate across geographic locations. Cultures have absorbed and integrated elements from foreign cultures for a long time. Moral ideas are not the property of one society, they belong to all humanity. Although the rejection of western moral imperialism comes from the justified assertion that the west has historically asserted moral superiority over its colonies and over the non-western world in general, it is not a sound reason to denounce bioethical principles using the misguided idea of “western, therefore, inapplicable in the east”.

We are seeing a rise in articles on Asian bioethics (Tai, 2008), Ibero-American bioethics (Pessini et al., 2010), Islamic bioethics (Shomali, 2008), and so on. De Castro argues that by trying to combat the “alleged” western ethical imperialism, people who want to have their own Asian bioethics might fall into the trap of trying to look for a common Asian identity, thereby imposing the very homogenization that they object to in the “imperialistic bioethics” – but a single Asian identity does not exist (Castro, 1999). By trying to reframe bioethics regionally people look for a way to preserve their identity, which would be useful if the identities were in some way homogenous, but they are not.

Similarly, as opposed to what some might be leading on with their east–west assertions, there is no such monolithic thing as “Western values” either, as Becker notes:

[T]here is no such thing as “the” Western values which would neatly define human practice in countries from the Urals to the Rocky Mountains. The “West” too is not a monolithic entity but embraces a variety of value-laden cultures and traditions. (Becker, 1995)

Often the problems that are outlined as specific to informed consent in eastern nations and in developing countries have nothing to do with the *idea* of informed consent and it also has little to do with it being a western concept. Take, for instance, the facts presented in the form of practical difficulties encountered while applying informed consent and the conclusion drawn by Schuman:

[R]esearchers were often forced to penetrate layer after layer of tribal hierarchy and corrupted bureaucracy in order to obtain informed consent. Sometimes they had to ask the village elders or the husbands of women participants first, or employ police escorts, or have tea and snacks, “regardless of the time it took.” Plus, they had to struggle with the fact that some subjects don’t have telephones, or permanent addresses, and may even be afraid to sign their names. their findings “demonstrate[d] the inadequacy and complexity of applying western-based concepts of informed consent to developing countries.” Far from a universal right, the informed consent requirement is a culturally contingent concept that is frequently alien to societies in the Third World. (Schuman, 2012)

It appears that Schuman here intends to imply that informed consent is a moral ideal which cannot (in the sense of being unfeasible) be applied in certain contexts since it clashes with other ideals, beliefs, or practices held by those living in such contexts. But note that corrupt bureaucracy, tribal hierarchy, gender disparities, employment of police escorts, or being offered tea and snacks are all social facts and none of these facts say anything about whether informed consent is right or wrong as a principle.

Contrary to Schuman’s conclusion, the fact that informed consent, autonomy, or another principle as applied in the west does not work well in a different context does not entail that a different version of the principle or underlying value behind a principle does not exist in this new context. Recent empirical literature has proven just that. For instance, one cultural psychology study with college students in the United States, Australia, Mexico, Venezuela, the Philippines, Malaysia, China, and Japan, rated the extent to which the needs of relatedness, autonomy, and competence were satisfied in various roles. The study showed that fulfilment of autonomy (which the study described as “the need to experience one’s behaviour as freely chosen and volitional, rather than imposed by external forces”) was equally important to the participants’ general hedonic (i.e. positive and negative affect) and eudaimonic (i.e. personal growth, meaningful purpose in one’s life) well-being (Church et al., 2013). Another study conducted on the elderly in China and France found that satisfaction of autonomy was considered a basic psychological need which facilitated self-directed motivation, which was considered essential to psychological well-being by both elderly Chinese and French (Tang et al., 2021). Further empirical studies have shown that the self-determination theory (SDT), which postulates the importance of three basic psychological needs (i.e. relatedness, autonomy, and competence) in promoting achievement and well-being, is generalizable and that autonomy, in particular, is equally important across east and west (Nalipay et al., 2020; Tang et al., 2021; Yu et al., 2018). The results of these studies have proven contrary to the beliefs of many cross-cultural researchers who cast doubt on the generalizability of certain values to non-western cultures.

The second objection to the cultural difference argument is that people often confuse the dominant *moral* justification of informed consent (as promoting individual autonomy) and informed consent as a practical guide in biomedical research. Informed consent is not all about protecting and promoting individual autonomy. Scholars have argued that informed consent’s primary justifications could include fostering trust between researchers and participants, preventing abusive conduct, and providing the assurance that participants will neither be deceived nor coerced (O’Neill, 2003). Different people in different parts of the world can accept moral norms for different reasons. For instance, under the Indian philosophy of *dharma* (the path of righteousness)*,* it would be the *dharma* of a doctor or researcher to engage in virtuous acts and to act with honour by being respectful to her patients or research subjects. In Hinduism, one must cultivate good virtues and do good deeds to be liberated from the cycle of rebirth. As Krishna advises Arjuna,

Arjuna, he does not suffer doom in this world or the next; any man who acts with honor cannot go the wrong way, my friend. Fallen in discipline, he reaches worlds made by his virtue, wherein he dwells for endless years, until he is reborn in a house of upright and noble men. (Bhagvad Gita, trans. Miller, 1986)

This is simply to show that the reason why someone would accept a principle like informed consent could have little to do with respecting individual autonomy, as generally understood, and could be based on other values that could be used to justify the principle.

But even if protection of autonomy is taken as the primary justification of informed consent, it still would not be an alien idea for non-western cultures. Autonomy has been conceptualized in many different ways and there have been several definitions that break away from autonomy as demonstrating some form of hyper-individualism (Dworkin, 1988; Jagger, 1983). Empirical studies, that have found no fundamental difference in the significance of autonomy across different cultures, depict a collectively-described experience of autonomy as a multidimensional space within which individuals vary across and within cultures, in ways that partly overlap and are partly unique (Littlewood, 1999). Moreover, there is also a relational conception of autonomy which takes into account how people define “self” in relation to others (Christman, 2004; Taylor, 2019), and it is this relational understanding of autonomy that can account for collectivist or communitarian decision-making that is exhibited in certain cultures (Behrens, 2018).

The cultural difference argument, when closely analyzed, does little to display a difference in eastern and western values, rather it shows that the globally (or widely) accepted or popularized formulations of notions like informed consent and autonomy cannot be adequately applied in societies where a significant portion of the population, institutions, etc., display some traits that cannot be accommodated by such narrow formulations. Stakeholders invested in bioethical debates must be able to distinguish between a moral principle, the reason to accept a moral principle, and the practice of a principle in a given society. For it appears that the proponents of the cultural difference argument often confuse the social facts that might impede the application of a principle with the justification or value of that principle.

The third objection to the “cultural difference argument” comes at the manner in which some scholars trivialize the important issues of uninformed consent, or lack of respect for individuals, or deceitful conduct by making it all about culture. Suggesting that bioethical principles are western-based and alien to societies in the third world, and thus inadequate, is grossly misleading. To take informed consent in the research context, the principle here is an attempt to rebalance the unequal power relationship between the researcher and the research subject. The researcher is reasonably assumed to have all the information (pertaining to a biomedical or health research study) and the research participant is reasonably assumed to have none. If information is withheld or misrepresented it could potentially threaten the life, health, and safety of the research participant. Informed consent tries to empower participants by giving them the right to make informed decisions about their health and well-being. Informed consent, as a non-ideal notion, is flexible enough to take into consideration the practical difficulties that could arise in obtaining consent from different population groups. Bioethical principles, like informed consent, are not about culture, east or west, north or south; they are about the protection of individuals and their interests regardless of what they believe and regardless of where they reside.

We might not have common cultures, traditions, or beliefs, but bioethical problems are common to all of us. What we face today, with the rise of rapid medico-technological advancements, poses challenges to all of us, not just as Asians, Americans, or Europeans, but as humans. While the recognition of diversity is an absolute necessity in the modern world, we might have been focusing on differences so much that they have come at the cost of recognizing the convergence of different values. As Amartya Sen points out:

The recognition of diversity within different cultures is extremely important in the contemporary world, since we are constantly bombarded by oversimple generalizations about “Western civilization,” “Asian values,” “African cultures,” and so on. These unfounded readings of history and civilization are not only intellectually shallow, they also add to the divisiveness of the world in which we live … The thesis of a grand dichotomy between Asian values and European values adds little to our comprehension, and much to the confusion about the normative basis of freedom and democracy. (Sen, 1997)

Situations requiring ethical decision-making nowadays are quite unlike those that the generations before us faced. For example, we are redefining the very idea of “personhood” because of advancements in technologies which manipulate basic biology through altered DNA, XNA, and proteins (Polkinghorne, 2004). We are also expanding the field of bioethics to include astroethics while assuming the possibility of life in space (Chon-Torres, 2018). But all these advancements are taking place alongside much of the local space battling social facts that place barriers in the pursuit of a virtuous life. Bioethical governance, therefore, requires a perspective that recognizes these challenges not along the east–west dichotomy but along an understanding of global/local interactions – a perspective that uncovers the ethos of different local contexts to find the global in it. Glocalization of bioethics, as will be discussed next, requires an admixture of local and global that is not simply lip service to a fusion that sounds catchy but is realistic in its pursuit of rightness amidst hard situations.

## Towards glocalization: bioethical governance and the Lesbian rule

The rise of international and multi-cultural research collaborations has generated a move towards ethical and legal standardization. The consolidation and institutionalization of bioethical principles and practices into advisory and regulatory structures on a global level – what we can call “global bioethical governance” – is part of such a move. Global bioethical governance has faced quite a few challenges in international and multi-country research collaborations – from outright ethical violation of informed consent norms to fabrication of data for research studies (Sleeboom-Faulkner & Patra, 2008). Many of these challenges with bioethical governance are said to result from a common root: the supposed *global* character of global bioethical governance. Critics often point out that the standardization of bioethical principles and practices prioritizes western ideals and favours their imposition upon local resource-poor medical settings (Po-Wah & Lai, 2002). It is thus argued that because of its western biases, global bioethical governance *cannot* adequately deal with the moral and ethical complexity that is found in non-western, cultures (Brody, 1997; Irvine et al., 2002). This notion has been challenged in recent empirical studies on bioethical governance.

For instance, a study from India showed that bioethical governance faces challenges because there are many competing factors (like time and funding constraints, bureaucratic red tape, easy to recruit vulnerable population groups, poverty and lack of access to healthcare, lack of or weak local ethical governance structures, scientific career concerns, etc.) at play in the day-to-day lives of the people on whom the principles are applied (whom I call “bioethical subjects”). The study showed that while most bioethical subjects appeared to appreciate the purpose of bioethical principles like informed consent, some acknowledged that owing to competing factors there could be apathy towards them (Bhakuni, 2019). These factors are a direct result of the social, economic, political, bureaucratic, and legal circumstances within a given society. The issue then is not entirely with the standardization of the principles and practices but mostly with the *application* of such standardized principles and practices. It is the local circumstances to which bioethical principles need to adapt for securing the bare minimum of the values that *all* societies cherish.

Given the challenges associated with local circumstances, global bioethical governance must internalize the process of glocalization, much along the lines of the Aristotelian metaphor for the importance of flexibility to achieve equitable justice – or the “Lesbian rule” – which suggests that

when the thing is indefinite the rule also is indefinite, like the leaden rule used in making the Lesbian moulding; the rule adapts itself to the shape of the stone and is not rigid, and so too the decree is adapted to the facts. (Aristotle ca. 350 B.C.E./2009)

Now, one might consider this to be a trivial point – that we should tailor the application of principles to context. But Aristotle’s point was not just that it is desirable to tailor principles to specific situations but that we should not think about morality and practical reasoning in terms of the application of general, unmalleable, principles or standards in the first place. For Aristotle, our practical sphere is like the rugged terrain: the Lesbian rule is the only way to measure it. While general principles can, and often do, work as guidelines for moral/practical reasoning, they are just that: guidelines. Under this conception, bioethical principles cannot then be understood as determinative but only as “contributory” – meaning that the principles are not themselves meant to tell us how to resolve a bioethical dilemma, but they leave us to determine the overall balance of right and wrong in it (Dancy, 2017). Aristotle's recommendation in not seeking greater precision in ethics than the subject-matter permits (e.g. in I.3, 1094b) also aligns with the idea of treating principles as contributory rather than determinative in bioethical decision-making. Note that even though I might be defending some weak version of moral particularism in this paper, the details of such particularist project are beyond the scope of this paper. I am only employing the Lesbian rule here to make a point about the importance of context to the kind of practical reasoning characteristic of bioethics governance.

I am not the first person to use the term “glocal” within the context of bioethics. In earlier works around glocal bioethics, the idea that values are necessarily in conflict in different socio-political contexts is widely accepted. In one glocal bioethics model developed around international Institutional Review Board (IRB) collaborations, scholars proposed a minimum threshold (based on a human rights approach) that cannot be crossed while negotiating basic human values (Dove & Ozdemir, 2014). But, under my proposal, bioethical governance does not require a negotiation of values. Each culture makes space for values like compassion, honesty, honour, integrity, humanity, etc. In Rachels’ seminal paper, *The Challenge of Cultural Relativism,* the notion that peoples’ cultural practices in different parts of the world show that different cultures have different moral values was methodically critiqued and debunked (Rachels, 2015). Rachels compared two different cultural practices involving treatment of the dead, where one group ate their dead and another buried their dead. When asked for reasons behind their different practices, there seemed to be no fundamental disagreement over the value of respecting the dead. The difference lay in the practice (or belief) of showing respect to the dead (Rachels, 2015). Fundamental bioethical values of doing no harm, respecting a human being, doing good to others, and fairness can be found across all cultures and societies (D. Brown, 1991; Chomsky, 2005). The manifestation of these values might be different in different contexts, therefore, the challenge for the field of bioethics is to find ways in which the commonality of values is established while allowing for the local manifestation of certain values to find its place in governance and practice.

Naturally, there will always be cases where some local manifestation of a value would contradict with another equally cherished value. But such contradictions can be found anywhere in the world and do not have to be defined along the east–west dichotomy. Such cases would correspondingly find disagreements between ethicists who claim to share the same values but come from different schools of thought. Similarly, disagreements can also be found over the prioritization of one human right over another. To talk of incommensurability between eastern and western values is an easy way out – a way to resolve moral conflicts and issues by saying that there is no way. Glocal bioethics does not conceal the fact that moral reasoning is hard, that disagreements exist, and moral principles and guidelines often do not give us all the answers. The way to solve bioethical problems involves thinking hard about the particularities of each case, by being pliant to pursue equitable justice or the best possible moral outcome in a given situation.

Any work towards glocalization of bioethics would require an understanding of how people in their capacity as patients, research participants, family members, researchers, health industry, and health care providers in different communities (and at different times) interpret illness, healing, and moral obligations. It would also require an understanding of local and indigenous connections between sentient biotic and non-sentient abiotic systems. We would require an enormous amount of collaborative social science research and theoretical reflection to work out the complex realities to which bioethical governance needs to respond in the real world. This becomes particularly important in international collaborations where researchers might be unfamiliar with the socio-economic, legal, political, and cultural settings.

But most importantly what glocalization of bioethics would require is the realization that abstract principles are played out in the lived experience of the responsibility for the other. Such responsibility is not only limited to people who owe duties under the regular bioethics literature but also include duties that are owed by the society at large. Just as a researcher has a responsibility for her research subject, the society of which the researcher forms a part has a responsibility for all its citizens to allocate and distribute the needed resources to meet fundamental human needs (Finkler, 2008). But despite a rapid increase in empirical bioethics studies, the theoretical bioethical literature largely ignores lived experiences of the bioethical subjects. A very large portion of bioethical subjects around the globe live in poverty and desperation. But since the loudest voices in bioethics come from high-income countries (HICs) the lived experiences of bioethical subjects in low – and middle-income countries (LMICs) are either ignored or are subjected to generalizations and misrepresentations.

Farmer’s critique in *Pathologies of Power* might still hold true for much of principle-oriented bioethics even today, he writes:

One gets the sense, in attending ethics rounds and reading the now-copious ethics literature, that these have-nots are an embarrassment to the ethicists, for the problems of poverty and racism and a lack of national health insurance figure only rarely in a literature dominated by endless discussions of brain death, organ transplantation, xenotransplantation, and care at the end of life. When the end of life comes early—from death in childbirth, say, or from tuberculosis or infantile diarrhea—the scandal is immeasurably greater, but silence reigns in the medical ethics literature. (Farmer, 2004)

His other critique that bioethics has been taken over by experts at the cost of obscuring the voices of those who have far more direct experience with issues requiring ethical decision-making still rings true. Bioethics, particularly the principle-oriented version, draws on the experience-distant field of philosophy which coupled with the HIC orientation of majority of bioethicists does not help to bring the kind of Lesbian rule flexibility that global bioethical governance requires. A Lesbian rule kind of flexibility would let bioethical subjects employ practical reason to tweak bioethical principles while making a moral decision in a particular situation. This would not leave bioethical decision-making solely in the hands of experts who do not have a full grasp over circumstances within which bioethical subjects take decisions. This, of course, is not being proposed to belittle the role of experts in bioethics, but to quote Churchill (1999):

Ethics, understood as the capacity to think critically about moral values and direct our actions in terms of such values, is a generic human capacity. Except for sociopaths, it is common to all of us, and skill in ethics does not lend itself easily to encapsulation in theoretical categories, core competencies, or a professional speciality.

Glocalization of bioethics cannot be achieved without a closer understanding of the lived realities of the bioethical subjects. Global bioethics will always be a space for experts, but to be truly *glocal* it also needs to make space for bioethical subjects to deliberate on what would be the appropriate methods to achieve rightness in each of their actions given their circumstances.

## Conclusion

In this paper, I argue that for the field of bioethics to redefine itself it must move away from the east–west dichotomy that appeals to some scholars. For that to happen, stakeholders in bioethical decision-making must be able to distinguish between a moral principle, the reason to accept a moral principle, and the practice of a principle in a given society. To tackle the issues that arise in the governance of global bioethics, I take inspiration from Aristotle’s metaphor about the Lesbian rule to propose glocalization of bioethics that not only aims for flexibility in the application of bioethical principles in local settings but also demands a deeper understanding of the lived experiences of bioethical subjects.
